# Attitude towards donation of the excised foreskin after circumcision surgery for research: A study from Madinah, Saudi Arabia

**DOI:** 10.1371/journal.pone.0293366

**Published:** 2023-10-24

**Authors:** Omar F. Khabour, Waleed H. Mahallawi, Aiman I. Ali, Hamdi H. Almaramhy, Abdulaziz M. Bakhsh, Ahmed Abu-Siniyeh

**Affiliations:** 1 Department of Medical Laboratory Sciences, Faculty of Applied Medical Sciences, Jordan University of Science and Technology, Irbid, Jordan; 2 Medical Laboratory Technology Department, College of Applied Medical Sciences, Taibah University, Madinah, Saudi Arabia; 3 Pediatric Surgery Division, Department of Surgery, College of Medicine, Taibah University, Madinah, Saudi Arabia; 4 Urology Department, College of Medicine, Taibah University, Madinah, Saudi Arabia; 5 Department of Clinical Laboratory Sciences, Faculty of Science, The University of Jordan, Amman, Jordan; University of Oklahoma Health Sciences Center, UNITED STATES

## Abstract

Studies have shown the possibility of using the part of the foreskin removed after circumcision in the field of scientific and therapeutic research. Donations of tissues and organs are always associated with ethical challenges posed by bioethicists and societies to ensure the appropriate use of these tissues/organs. The purpose of this study was to understand the attitudes and awareness of parents/guardians regarding donation of excised foreskin to research and medical use. The study was based on a questionnaire and included 133 parents/guardians who visited Uhud Children’s Hospital in Madinah, Saudi Arabia for newborn male circumcision. The results showed a high willingness (61.7%) to donate the extracted foreskin to research. The willingness to donate the extracted foreskin to research associated with undergraduate degree (P = 0.018), male sex (P = 0.011), high income (P = 0.029), and participation in previous research studies (P = 0.002). About 41.8% were convinced that written informed consent should be obtained before circumcision surgery, 38.1% (n = 51) were convinced that written informed consent should be taken after surgery, while the remaining 19.4% reported that the timing of written informed consent is unimportant. Finally, fear of excision of excess tissue (74.5%), lack of confidence in the research (68.6%), and potential for commercial use (64.7%) were the main barriers to unwillingness to donate the excised foreskin for research. In conclusion, a reasonable portion of Saudis agreed to donate their foreskin for research purposes. There is an urgent need to enhance awareness and attitudes towards tissue donation for research and therapeutic use.

## Introduction

The foreskin is defined as a continuation of skin from the shaft of the penis that covers the urethral meatus and glans penis [1]. In male circumcision, the foreskin is surgically removed from the penis. Circumcision is one of the most common practices around the world, which is routinely performed to adhere to the teaching of Prophet Abraham. There is a general belief that performing such a practice does not violate human integrity nor cause any kind of moral inadequacy [2].

Foreskin cells are a source for embryonic stem cells. Foreskin cells have shown the ability to develop into a stable cell line of any of the three embryonic germ layers under in vitro conditions [3, 4]. In addition, foreskin cells have been shown to be useful as a model for studying cellular damage and mitochondrial disfunction [5, 6]. Moreover, the foreskin has been shown to be a good source for mesenchymal stromal cells that have several immunotherapeutic applications [7–9]. Finally, stem cells derived from foreskin have diverse therapeutic potential for many conditions including periodontal diseases, skin damage, and neurodegenerative diseases [10–14].

The medical importance of male circumcision has created a vital opportunity for bioethicists and medicinal parties to define the moral significance of circumcision through the principles of bioethics context [15]. In a Korean study, authors encouraged further clinical research on the biological description of the foreskin. The study also recommended that the potential benefits and disadvantages of circumcision be disclosed to the parents [16]. By assessing the parents’ knowledge, the recommendation came to demand the improvement of education, circumcision culture and foreskin issues in society [17]. Thus, it is important to understand the attitude and perception of the population towards donations of embryonic stem cells for research and medical use [18–20]. Therefore, this study aimed to investigate parents’ perception of donating foreskin for research and its beneficial influences in the therapeutic field.

## Methods

### The study participants

The study is quantitative and cross-sectional in design. The study procedure was approved by the Ethics Committee of the Faculty of Applied Medical Sciences, Taibah University, Saudi Arabia (Ref No. MLT 2021–050). Parents/guardians who visited Children Hospital in Madinah city/Saudi Arabia for newborn male circumcision were invited to participate in the study. A convenient (non-probabilistic) sampling approach was adopted. Adult parents/guardians who visited the circumcision clinic were invited to volunteer in the study. Sample size was estimated using G-Power 3.1 (Universitat Kiel/Germany) based on a medium effect size, an alpha of 0.05 and a power of 0.95. The minimum required number of subjects was 111. A total of 200 subjects were invited to participate, of whom 133 volunteered in the study. The response rate was 66.5%. Informed consent was taken from all participants after a full explanation of the study goals.

### The study instrument

The study questionnaire was developed based on previous studies that examined people’s attitudes toward donating tissue for research [21–23]. The first part of the instrument asked questions about general demographics. This includes age (stratified at approximately 10-year intervals), gender (male, female), education (<high school, high school, diploma, and university degree), place of living (city vs. village), income, and history of participation in previous studies (yes vs. no). Household income in Saudi Arabia Riyale (SAR) was divided into low (<7000), medium (>7000–15000), and high (>15000). The second domain asked participants about their willingness to donate the excised foreskin for research. This was inferred by the question: Are you willing to donate the excised foreskin for research? The given choices were “Yes” and “No”. As for the third domain, we asked about the reasons for refusing to donate the excised foreskin for research. Participants were presented with five items with a choice of “Yes” or “No”. The items were: lack of trust in research, potential for non-research use of tissue, fear of excision more tissues than it should be, potential for commercial use and conflict of ownership. The instrument was revised by three experts in the field and pilot tested on 20 participants. The final version of the instrument was prepared and administered using “Google Forms” and filled out using a tablet. Data were collected during the period from April 2021 to October 2021. The collected data was accessed and processed during the first quarter of 2022. Study data is available as S1 File.

### Statistical analysis

Statistical analysis was computed using SPSS software, version 21. Demographic variables were expressed as frequencies and percentages. A binary regression model was used to analyze factors associated with willingness to donate foreskin for research. An alpha of less than 0.05 was used as the threshold for statistical differences.

## Results

The study included 133 parents/guardians who visited Children’s Hospital in Madinah, Saudi Arabia, for newborn male circumcision. Participant demographics are shown in Table 1. The majority were males (55.6%), living in the city (68.4%), less than 50 years old (76.7%) and had not previously volunteered in research (54.9%). Less than half (48.9%) have an income less than 7000 SAR and about a third have a university degree. About a quarter of the participants (n = 36, 27.1%) believed that they own the excised foreskin of their newborn. Of the sample, 82 (61.7%) agreed to donate the excised foreskin after circumcision surgery for research purposes.

**Table 1:** Characteristics of the participants.

Item	Sub-item	N = 133 (%)
**Age groups**	18–30	40 (30.1)
	31–40	30 (22.6)
	41–50	32 (24.1)
	>50	31 (23.3)
**Education**	< High school	42 (31.6)
	High school	33 (24.8)
	Diploma	17 (12.8)
	University degree	41 (30.8)
**Gender**	Male	74 (55.6)
	Female	59 (44.4)
**Income (Saudi Riyal)**	<7000	65 (48.9)
	7000–15000	50 (37.6)
	>15000	18 (13.5)
**Place of living**	City	91 (68.4)
	Village	42 (31.6)
**Prior research participation**	Yes	60 (45.1)
	No	73 (54.9)
**willingness to donate excised foreskin for research**	Yes	82 (61.7)
	No	51 (38.3)

Table 2 shows a cross tabulation of demographic factors with willingness to donate excised foreskin for research. The results showed significant associations between willingness to donate foreskin tissue for research and education, gender, income, and previous research participation. Higher willingness to donate associated with university degree (P = 0.018), male gender (P = 0.011), high income (P = 0.029), and prior research participation (P = 0.002). Binary regression analysis (Table 3) showed that prior research participation was related to the willingness to donate foreskin for the research (odd ratio: 0.257 and 95% C.I. [0.115–0.575]).

**Table 2:** Cross-tabulation of demographics of participants with agreeing to donate excised foreskin to research.

Item	Sub-category	Agree to donate	Disagree to donate	P value
**Age groups**	18–30	24 (60.0)	16 (40.0)	0.357
	31–40	21 (70.0)	9 (30.0)	
	41–50	16 (50.0)	16 (50.0)	
	>50	21 (67.7)	10 (32.3)	
**Education**	< high school	23 (54.8)	19 (45.2)	0.018
	High school	18 (54.5)	15 (45.5)	
	Diploma	9 (52.9)	8 (47.1)	
	University degree	32 (78.0)	9 (22.0)	
**Gender**	Male	52 (70.3)	22 (29.7)	0.011
	Female	30 (50.8)	29 (49.2)	
**Income (Saudi Riyal)**	<7000	34 (52.3)	31 (47.4)	0.029
	7000–15000	35 (70.0)	15 (30.0)	
	>15000	13 (72.2)	5 (27.8)	
**Place of living**	City	60 (65.9)	31 (34.1)	0.097
	Village	22 (52.4)	20 (47.6)	
**Previous research participation**	Yes	53 (72.6)	20 (27.4)	0.002
	No	29 (48.3)	31 (51.7)	

**Table 3:** Binary regression analysis of factors that affect donation of excised foreskin to research.

Item	B	P-value	OR (95% C.I.)
**Constant**	1.478	0.234	4.383
**Age**	-0.171	0.392	0.843 (0.569–1.247)
**Education**	-0.224	0.291	0.799 (0.527–1.212)
**Sex**	0.709	0.074	2.032 (0.934–4.423)
**Income**	-0.462	0,222	0.630 (0.300–1.323)
**Place of living**	0.571	0.224	1.771 (0.705–4.445)
**Prior research participation**	-1.358	0.001	0.257 (0.115–0.575)

Participants were asked about the informed consent process for the donation of the excised foreskin for research. About 41.8% (n = 56), believed that written informed consent should be taken before circumcision surgery, while 38.1% (n = 51) believed that written informed consent should be taken after surgery. On the hand, the rest 19.4% (n = 26) reported that the timing of written informed consent is not important.

Participants were asked about applications of excised foreskin tissue. Most of the participants agreed on the use of excised tissue in environmental research (85.0%), genetic testing (83.5%), toxicological studies (82.7%), vaccine research (86.5%), and stem cells therapeutic research (86.5%).

Table 4 showed the reasons behind the unwillingness to donate the excised foreskin for research. Fear of excision of extra tissues (74.5%), lack of trust in the research (68.6%), and potential of commercial use (64.7%) were the main reasons behind such unwillingness.

**Table 4:** Reasons for refusing donating foreskin for research.

Reason	Yes	No
Lack of trust in research	35 (68.6)	16 (31.4)
Potential for non-research use of tissue	17 (33.3)	34 (66.7)
Fear of excision more tissues than it should be	38 (74.5)	13 (25.5)
Potential for commercial use	33 (64.7)	18 (35.3)
Conflict of ownership	10 (19.6)	41 (80.4)

## Discussion

In the current study, the majority of participants (61.7%) agreed to donate excised foreskin from their newborn sons after circumcision surgery for research purposes. In addition, they agreed on the use of excised tissue in environmental research, genetic testing, toxicological studies, vaccine research, and stem cell research. The usual procedure for managing the extracted foreskin is to dispose of it in the biological waste, but some studies have revealed the huge potential of foreskin cells as an important resource for medical research. It has been shown that the foreskin contains a variety of valuable cells including fibroblasts, the primary cell that combines connective tissue. It also acts as an important pathogenic/diagnostic model of microorganisms and as a model for the examination of medications [24]. Several studies revealed that human foreskin cells could generate stem cells for tissue repair and renewal, and for the treatment of diseases [11, 25, 26].

Willingness to donate tissue for research has been examined by several studies from different parts of the world. In the current study about two-thirds of the participants were willing to donate the excised foreskin for research. This finding is consistent with a previous study conducted in Saudi Arabia that examined the public’s willingness to participate in a dental biorepository for research purposes [27]. In a study of donors from the Portuguese Public Bank of Gametes donors, the majority agreed to donate their gametes for research [28]. In a study from Germany, about 77% of the participants agreed to donate their eyes for research after death [29]. Similarly, about 77% of immigrant population in the USA reported a willingness to donate tissue for research purposes [30]. The same level of willingness to donate tissue for research has been reported was in countries such as Canada, Sweden, Nigeria, and Jordan [31–34]. In Poland, the willingness to donate tissue for research has been to vary according to tissue type ranging from 74% for urine samples to 20% for post-mortem brain fragments [35]. According to previous literature most populations were positive about tissue donation for research.

Willingness to donate foreskin tissue for research was found to be associated with educational level (university degree), male gender, higher income, and previous research participation. In a study conducted in Saudi Arabia, education, female gender, previous participation in medical research, and higher income were found to be associated with tissue donations for dental research [27]. Thus, the current findings are consistent with what was previously reported in Saudi Arabia. Gender difference in willingness to donate gametes for research has been reported in a study conducted in Portugal [28]. Regarding income, studies have shown that individuals with higher economic status were more positive towards tissue donation and toward participation in cancer clinical trials [36–38]. Educated people and those with experience in research participation are expected to be more aware of the importance of research in advancing knowledge and healthcare. People of lower economic status may believe that tissue donation may add an additional financial burden that they cannot afford. More research is needed to evaluate such speculations. Specific factors associated with willingness to donate tissue for research can be used through educational interventions to encourage tissue donation for research in the country.

Nearly more than a third of the participants (41.8%) deemed that written informed consent to donate the excised foreskin for research should be taken before the circumcision surgery, while 38.1% believed that consent should be taken after the surgery. On the other hand, the rest were neutral regarding the timing of informed consent. If the foreskin is donated for research, the excised foreskin may need to be transferred directly to appropriate media to maintain the health of the donated tissue. However, measures should be taken to avoid excision of excess foreskin tissues beyond the recommended if parents/guardian consent to tissues donation. It is worth to mentions that several studies in the MENA region, including Saudi Arabia, were reviewed, and discussed the importance of informed consent and its applicability in this region. Issues of culture, religion, and lack of legislation were among the challenges of the informed consent in the MENA region [39–41].

In the current study, among the reasons underlying refusal to donate the excised foreskin for research were fear of excision of excess tissue, lack of trust, and possibilities of commercial use. Trust has been reported by several studies to be crucial for tissue donation and participation in research and clinical trials [28, 42, 43]. For example, a study that was conducted in Poland showed that the willingness to donate tissue for research was mostly shaped by social trust in physicians and scientists [35]. The possibility of commercial use can also shape the willingness for research participation [44–46]. For example, in a study that examined the willingness of the Swiss public to participate and donate tissue to biobanks, the potential for misuse of donated tissue for commercial or marketing purpose was among the determinants of the willingness [46]. Thus, taking such determinants into account may enhance tissue donation for research. Implementation of educational interventions can greatly enhance population awareness of tissue donation for research and clinical use [47].

Among the limitations of the current investigation is that the study was conducted in one city in Saudi Arabia. It is recommended in future studies to expand the study to include other cities in the Kingdom. This cross-sectional study adopted a qualitative approach. Adopting a qualitative approach could in the future explore in depth the reasons behind unwillingness to donate tissue for research.

## Conclusion

A satisfactory segment of the Saudi population was positive regarding donation of the foreskin for research. This positive attitude was associated with the education level, income, and previous research participation. Educational interventions can enhance the willingness to tissue donations and participation in scientific research.

## Supporting information

S1 File (XLSX)
